# Impact of a diabetes screening program on a rural Chinese population: a 3-year follow-up study

**DOI:** 10.1186/s12889-015-1570-3

**Published:** 2015-02-27

**Authors:** Yanlei Zhang, Feng Ning, Jianping Sun, Zengchang Pang, Xiaoyong Wang, Anil Kapur, Harri Sintonen, Qing Qiao

**Affiliations:** Department of Public Health, Clinicum, University of Helsinki, Mannerheimintie 172, PL41, Helsinki, 00014 Finland; Qingdao Municipal Centre for Disease Control and Prevention, Qingdao, China; Provincial Hospital Affiliated to Shandong University, Jinan, Shandong China; World Diabetes Foundation, Gentofte, Denmark; Observational Research Center, GMA, AstraZeneca, Mölndal, Sweden

**Keywords:** Screening, Pre-diabetes, Health-related quality of life, Depression, Lifestyle

## Abstract

**Background:**

Screening for type 2 diabetes helps detect previously unknown diabetes and identify people with pre-diabetes, but the adverse impact of such screening on individuals labelled as pre-diabetes or classified as normal, is less known. In this study the health-related quality of life (HRQoL), depression and lifestyle changes in a rural Chinese population are assessed three years after a screening program.

**Methods:**

A total of 647 (39.1%) individuals with pre-diabetes and 1009 (60.9%) individuals with normoglycaemia from a population-based diabetes screening program in 2009 were re-examined in 2012–2013. Changes at the end of 3 years in HRQoL, depression, BMI, weight, frequency of physical activity and vegetable intake were assessed.

**Results:**

In men with normoglycaemia the mean (SD) 15D scores were 0.974 (0.04) at baseline and 0.973 (0.05) at follow-up; and 0.971 (0.05) and 0.966 (0.06) for men with pre-diabetes. In women the scores were 0.973 (0.05) and 0.963 (0.06) for normoglycaemia and 0.959 (0.06) and 0.954 (0.07) for pre-diabetes, respectively. Compared to baseline, the HRQoL was slightly lower at 3 years in all groups but the change was not considered to be clinically important, and was only statistically significant for women with normoglycaemia (p < 0.05). The depression score was slightly elevated in women, but not in men. No significant changes in BMI were noticed, but weight increased slightly in the normoglycemia group (p < 0.05). Screening had a significant positive impact on physical activity and vegetable intake.

**Conclusions:**

This population-based diabetes screening program generated long-term positive changes toward a healthy lifestyle as measured by physical activity and vegetable intake for all the participants without adverse effects on the HRQoL and depression.

## Background

Diabetes is becoming one of the most significant public health concerns in China. Based on two consecutive population-based diabetes surveys in Qingdao, China, the age-standardized prevalence of diabetes and pre-diabetes amongst adults aged 35–74 increased from 12.2% and 15.4% in 2002 to 18.8% and 28.7% in 2006 in urban areas, and 14.1% and 20.2% in rural areas in 2006 [1]. A national study among Chinese adults between June 2007 and May 2008 reported that the age-standardized prevalence of diabetes and pre-diabetes were 9.7% (10.6% among men and 8.8% among women) and 15.5% (16.1% among men and 14.9% among women), respectively [2]. In the 2010 China non-communicable disease national survey, the prevalence of diabetes and pre-diabetes was estimated to be 11.6% and 50.1% in the Chinese adult population [3]. Diabetes comorbidities and late complications not only link to the increase morbidity and mortality, but also adversely effect on the quality of life of the patients, and impose a heavy economic burden on the healthcare system [4].

In order to identify people with diabetes early and initiate treatment to prevent progression screening for undiagnosed diabetes has been advocated in several countries. Such screening programs can also identify individuals with pre-diabetes who are at high risk for diabetes and may benefit from early intervention [5-7]. However in asymptomatic people, screening may create psychological stress in individuals labelled as having diabetes or pre-diabetes [8,9], and a false health assurance signal amongst those labelled normal. The potential false reassurance may decrease intentions for behavioural change, keep unhealthy behaviours or delay in seeking medical help [10,11]. No published studies have addressed these issues amongst the Chinese population. The aim of our study was to assess changes in health-related quality of life (HRQoL), depression and lifestyle amongst a Chinese rural population who underwent a screening program three years ago and were labelled either as having pre-diabetes or normoglycaemia at baseline.

## Methods

### Study population

A population-based diabetes survey was conducted in 2009 in three rural regions (Jimo, Huangdao, Jiaonan) in Qingdao, China. A stratified, random cluster sampling method was used to recruit a representative sample of the general rural population aged 35–74 years living in selected villages for at least 5 years. A total of 5556 individuals living in selected villages were invited and 3757 individuals participated in the survey, giving a response rate of 67.6%. From June 2012 to May 2013, a total of 3108 participants who did not have diabetes at baseline screening were invited for re-examination. Of these, 1782 individuals attended the second examination (follow-up rate 57.3%), with 1656 participants provided complete information for the current data analysis.

### Screening methods

After an overnight fast, all the participants without previously diagnosed diabetes underwent a standard 75 g oral glucose tolerance test (OGTT) from 07:00 to 11:30 h at the local survey sites, and blood samples were collected from the antecubital vein into a vacuum tube containing sodium fluoride. Plasma glucose was determined by the glucose oxidase method. All participants received their glucose test results one or two months after the survey.

The surveys were approved by Qingdao Municipal Health Bureau and ethics committee of the Qingdao Municipal Center For Disease Control and Prevention. Written consent was obtained from all participants before the surveys.

In both examinations, all the participants were interviewed at the local survey sites by a trained survey team consisting of doctors and nurses. Family history of diabetes was defined as at least one first degree relative (parents, siblings or offspring) having diabetes. Education was classified as illiterate, elementary school (1–6 years), junior high school (7–9 years) and high school or above (≥9 years formal education). Personal monthly income was classified as income < ¥300/month, ¥300-¥999/month, ≥¥1000/month (1 euro ≈ 7.2 chinese yuan¥). Height and weight were measured with participants wearing only light clothes and without shoes. For surveys carried out in winter, 1 or 2 kilogram was subtracted from the measured weight of individuals depending on the woolens they wore. Body mass index (BMI) was calculated as weight in kilograms divided by height in meter squared (kg/m^2^). Waist circumference was measured at the mid-point between the rib cage and the iliac crest to the nearest 0.1 cm. All information was recorded on standard paper-based questionnaires during the interview both at baseline and follow-up. All the participants received health tips from the survey doctors or nurses during the interview and at the time when they were contacted again with their screening test results. No additional education sessions have been provided to the participants by the survey team.

### HRQoL measurement

We used an interview-based 15D instrument to measure the overall HRQoL at baseline and follow-up [12]. The 15D questionnaire consists of 15 dimensions: mobility, vision, hearing, breathing, sleeping, eating, speech, excretion, usual activities, mental function, discomfort and symptoms, depression, distress, vitality and sexual activity. Each dimension is divided into five ordinal levels, by which more or less the attribute is distinguished. The single index score (15D score), representing the overall HRQoL on a 0–1 scale (1 = full health, 0 = being dead) are calculated from the health state descriptive system using a set of population-based preference or utility weights. The minimal clinically important change or difference in the 15D score is ±0.015 [13].

The 15D questionnaire usually takes 5–10 minutes to complete. A Chinese version of the 15D questionnaire was translated according to the established international guidelines and in consultation with the original 15D developer (Harri Sintonen) and validated in Chinese individuals with a range of glucose tolerance status before it was administrated to the survey participants and also used in other published study [4].

The changes in HRQoL in the pre-diabetes and normoglycaemia group was assessed for the entire group, as well as for a subgroup who did not develop diabetes at 3 years in order to exclude the potential influence of the diabetes status.

### Depressive symptom measurement

The Zung self-rating depression scale (ZSDS) questionnaire was used to survey depressive symptoms [14]. The scale consists of 20 items using a four-point-grading system, ranging from “none or a little of the time” to “most of or all the time”. The completed questionnaire score ranges from 20 to 80. Individuals were classified into four groups based on their ZSDS scores: 20–44 normal range, 45–59 mildly depressed, 60–69 moderately depressed, and ≥70 severely depressed.

### Lifestyle measures

To evaluate the individual physical activity (PA), daily walk steps were counted using a questionnaire recommend in Physical Activity Guideline for Chinese Adults [15] by Ministry of Health in China. The questionnaire investigated five major types of daily physical activity patterns of Chinese adults: walking, housework, leisure time PA, cycling, and occupational PA. Four types of daily physical activity patterns except for occupational PA were considered in the current study. Each type of PA includes specific activities such as walking, cooking, taking care of children or group dancing etc. that are most common among Chinese adults. Participants reported the minutes per day of PA and frequency per week of each specific activity during the past week. Each specific activity based on the total amount and intensity was then translated into walk steps. The calculation was based on the Compendium of Physical Activities [16], which is a coding scheme that classifies specific PA by rate of energy expenditure. The energy expenditure of 1000 walk steps was equal to walking at 4 km/hour (METs = 3) for 10 minutes (1000 steps = 1/2 MET-hr). During the data analysis, physical activity level was arbitrarily classified as low (<6000 steps per day), moderate (6000–9999 steps per day), and high (≥10 000 steps per day).

The consumption of fresh vegetable was evaluated by an interviewer-administered 54-item food quantitative frequency questionnaire [17] that incorporated 54 kinds of food, beverage and other items that are commonly consumed in China. Participants reported the intake frequency and amount of fresh vegetables during the past year on a daily, weekly, monthly or yearly basis. Participants were classified into three categories based on fresh vegetable consumption of <7 times, 7–13 times, and ≥14 times per week.

Differences in the ZSDS score, BMI, weight, waist circumference, frequency in physical activity and fresh vegetable consumption between baseline and follow-up were calculated for pre-diabetes and normoglycaemia categories classified according to the baseline screening glucose tolerance test results. The baseline HRQoL, ZSDS and lifestyle measures were recorded before the participants received their glucose test results.

### Classifications of pre-diabetes and normoglycaemia

Individuals who were free of diabetes at baseline were further classified into pre-diabetes and normoglycaemia according to the World Health Organization/International Diabetes Federation 2006 criteria [18]. Pre-diabetes was defined if a baseline fasting plasma glucose (FPG) fell between 6.1 and 6.9 mmol/l and/or 2 h plasma glucose (2hPG) between 7.8 and 11.0 mmol/l; normoglycaemia was defined if a FPG < 6.1 mmol/l and 2hPG < 7.8 mmol/l. Diabetes was defined as FPG ≥ 7.0 mmol/l and/or 2hPG ≥11.1 mmol/l. Individuals with diabetes at baseline including those with a prior history of diabetes were not included in the current data analysis.

### Statistical analysis

Changes over time in the continuous variables (HRQoL, ZSDS, BMI, weight) within each group were analysed using the paired Student’s t test and in categorical variables (Physical activity, vegetable intake) using the McNemar’s test (binary) or Marginal homogeneity test (multinomial), respectively.

Differences of changes between the two groups were determined with analysis of co-variance (ANCOVA), adjusting for the corresponding baseline values of HRQoL, ZSDS, BMI and weight. The statistical analysis was performed using PASW statistics (Version 18.0.2, Chicago: SPSS Inc; April 2, 2010). Two-sided p-values <0.05 were considered statistically significant.

## Results

Among the participants at baseline, 39.1% (647/1656) individuals had pre-diabetes. The baseline demographic and clinical characteristics of the population according to the glucose test results at baseline are shown in Table 1. Amongst non-diabetic population, 21 individuals had developed diabetes during the follow-up period, and 124 individuals were detected as diabetes based on their glucose test results at the follow-up re-examination, with a 3-years cumulative incidence of diabetes of 8.8%. The incidence rate of type 2 diabetes was higher in individuals with pre-diabetes (16.1%) than in those with normoglycaemia (4.0%) at baseline. People with pre-diabetes were older and had significantly higher BMI, blood pressure, total cholesterol, triglycerides than those with normoglycaemia at baseline.

**Table 1:** **Baseline demographic and clinical characteristics of participants who were free of diabetes at baseline**

	**Normoglycaemia**	**Pre-diabetes**	p value*
No.	1009 (60.9)	647 (39.1)	
Diabetes at follow-up no. (%)	41(4.0)	104(16.1)	<0.001
Age (years)	50 (10.2)	54 (10.4)	<0.001
Men (%)	37.3	42.0	>0.05
Family history of diabetes (%)	4.5	6.5	>0.05
BMI (kg/m^2^)	24.5 (3.3)	25.5 (3.7)	<0.05
Personal income level (%)			
<300¥	43.8	44.8	>0.05
300-999¥	40.2	39.6	
≥1000¥	15.9	15.6	
School years (%)			<0.001
Illiterate	19.2	26.4	
Elementary school (1–6 years)	33.3	37.7	
Junior high school (7–9 years)	39.5	30.1	
high school or above (≥9 years)	7.9	5.9	
Systolic blood pressure (mmHg)	131 (20)	140 (22.4)	<0.001
Diastolic blood pressure (mmHg)	80 (11.6)	84 (11.7)	<0.001
Total cholesterol (mmol/l)	5.0 (0.9)	5.4 (1.0)	<0.001
Triglycerides (mmol/l)	1.2 (0.8)	1.5 (1.0)	<0.001
HDL-C (mmol/l)			
men	1.6 (0.4)	1.7 (0.5)	<0.05
women	1.7 (0.3)	1.6 (0.3)	<0.05

Data are age-adjusted mean (standard deviation) or percentage. *between normoglycaemia and pre-diabetes group; HDL-C, high-density lipoprotein cholesterol; BMI, body mass index.

### Changes from baseline to follow-up

At 3 years follow up screening, the HRQoL had slightly decreased in both groups but the change was not clinically important, and was statistically significantly only for women with normoglycaemia. After excluding patients diagnosed with diabetes at follow-up examination, the adjusted-mean of 15D score and changes between baseline and follow-up were no altered (Table 2).

**Table 2:** **Changes in HRQoL, depression and lifestyle variables between baseline and follow-up examinations**

	**All (n = 648)**			**Normoglycaemia (n = 376)**			**Pre-diabetes (n = 272) ‡**		
**Men**	**Baseline**	**Follow-up**	**Δ (SD)**	**Baseline**	**Follow-up**	**Δ (SD)**	**Baseline**	**Follow-up**	**Δ (SD)**
HRQoL*	0.973 (0.05)	0.970 (0.06)	−0.003 (0.07)	0.974 (0.04)	0.973 (0.05)	−0.001 (0.06)	0.971 (0.05)	0.966 (0.06)	−0.005 (0.08)
ZSDS	29 (7.8)	30 (7.1)	0.5 (10.3)	29 (7.7)	29 (7.1)	0.5 (10.1))	29 (7.9)	30 (7.1)	0.6 (10.6)
BMI (kg/m^2^)	24.4 (3.4)	24.5 (3.5)	0.1 (2.0)	24.1 (3.1)	24.2 (3.4)	0.2 (1.9)	24.9 (3.7)	24.9 (3.8)	0.04 (2.3)
Weight (kg)	68.1 (11.1)	68.5 (11.8)	0.4 (5.0)†	67.2 (10.5)	67.8 (11.3)	0.6 (4.5)†	69.3 (11.9)	69.6 (12.3)	0.2 (5.7)
Physical activity (%)									
Low	63.7	56.3†		61.7	59.8†		66.5	51.5†	
Moderate	22.8	16.5		22.1	14.1		23.9	19.9	
High	13.4	27.2		16.2	26.1		9.6	28.7	
Vegetable intake									
(times/week, %)									
<14	28.7	31.2		26.6	30.9		31.6	31.6	
≥14	71.3	68.8		73.4	69.1		68.4	68.4	
	**All (n = 1008)**			**Normoglycaemia (n = 633)**			**Pre-diabetes (n = 375) ‡**		
**Women**	**Baseline**	**Follow-up**	**Δ (SD)**	**Baseline**	**Follow-up**	**Δ (SD)**	**Baseline**	**Follow-up**	**Δ (SD)**
HRQoL*	0.968 (0.06)	0.959 (0.07)	−0.009 (0.08)†	0.973 (0.05)	0.963 (0.06)	−0.010 (0.07)†	0.959 (0.06)	0.954 (0.07)	−0.005 (0.09)
ZSDS	30 (7.9)	31 (7.2)	1.6 (10.5)†	29 (8.0)	31 (7.2)	1.6 (10.4)†	30 (7.7)	32 (7.1)	1.7 (10.7)†
BMI (kg/m^2^)	25.2 (3.5)	25.3 (3.7)	0.1 (2.1)	24.7 (3.4)	24.9 (3.4)	0.1 (1.9)	26.0 (3.6)	26.0 (4.0)	−0.03 (2.4)
Weight (kg)	62.1 (9.6)	62.4 (9.8)	0.4 (4.2)†	61.4 (9.4)	62.0 (9.8)	0.6 (4.1)†	63.2 (9.8)	63.2 (10.0)	−0.1 (4.4)
Physical activity (%)									
Low	34.9	13.4†		34.1	16.1†		36.3	17.3†	
Moderate	11.6	22.8		11.5	15.6		11.7	17.1	
High	53.5	63.7		54.3	68.2		52.0	65.6	
Vegetable intake (times/week, %)									
<14	34.1	25.5†		32.9	24.5†		36.3	27.2†	
≥14	65.9	74.5		67.1	75.5		63.7	72.8	

Data are presented as age-adjusted means(SD) or percentage unless otherwise indicated; HRQoL, health-related quality of life; ZSDS, zung self-rating depression scale; BMI, body mass index; SD, standard deviation.

*Adjusted for baseline age, BMI, living district.

†p < 0.05 between baseline and follow-up using paired t test for continuous variables, McNemar or Marginal homogeneity test for proportion within group.

‡p > 0.05 for all the changes between normoglycaemia and pre-diabetes group, with adjustment for baseline corresponding values of HRQoL, ZSDS, BMI and weight, using ANCOVA.

The ZSDS score was slightly higher at follow-up in both nomorglycaemia and pre-diabetes women, but not in men. No significant change in the BMI was noted in both groups, but weight increased slightly in the normoglycaemia group (p < 0.05). Physical activity increased in both men and women, but an increase in frequency of vegetable intake was observed in women only (Table 2).

## Discussion

To the best of our knowledge, this is the first population-based study to use preference-based instrument to evaluate the impact of a diabetes screening program on individuals who were labelled as pre-diabetes or normoglycaemia. Screening and labelling as pre-diabetes or normoglycaemia generated long-term positive changes toward a healthy lifestyle but had no adverse impact on the overall HRQoL and depression.

Our findings are consistent with the earlier studies in other countries which all underline the minimal, if any, adverse effect of screening with respect to HRQoL. A study among the general Dutch population indicated that new diagnosis of type 2 diabetes has no adverse or positive effect on the overall HRQoL, physical and emotional state, compared with non-diabetic subjects at 2 weeks, 6 and 12 months follow-up [19]. Edelman et al. showed that screening for type 2 diabetes has a minimal labelling effect on patients’ health status measured by SF-36 instrument 1 year after diagnosis [20]. The Diabetes Prevention Program (DPP) outcome study in US indicated that the participants with high risk of type 2 diabetes reported a decline in overall health status and overall physical HRQoL regardless of whether or not they were diagnosed as diabetes during the follow-up, but there was no change in the overall mental HRQoL [21]. The Anglo-Danish-Dutch study (ADDITION) investigated the psychological impact of a diabetes screening program on screening participants and non-invited controls. The study found that the screening program had a limited adverse psychological impact on screening attenders after initial blood glucose test, at 3–6 months and 12–15 months after screening as compared with non-invited controls [22].

Since the majority of individuals (60.9%) in our study had normoglycaemia, it is very important to estimate whether or not this generated a sense of false reassurance and encouraged continuation or even initiation of an unhealthy lifestyle (certificate of health effect). A previous study by Paddison et al. has shown that a negative screening result does not lower participants’ intentions to reduce dietary fat and sugar intake and to increase physical activity after first appointment or at 3–6 months or 12–15 months [23]. Willems et al. found that neither a negative screening test for diabetes nor non-invitation to a screening test made any difference to overt changes towards a healthier lifestyle at 4 years follow-up [24]. Our study found that screening for diabetes brought long-term positive lifestyle change in both -in the pre-diabetes and normoglycaemia groups equally. The diabetes screening surveys were carried out in parallel with Qingdao Diabetes Prevention Program [25], which is a lifestyle intervention program for diabetes covering certain areas of Qingdao city. Many diabetes education and health promotion activities through print and audio visual media; internet; free distribution of information booklets and diabetes risk score flyers, were carried out targeting the entire 1.94 million population living in the project areas. Additional education sessions were also provided to the identified high-risk population using a diabetes risk score [26]. These activities may have to some extent, contributed to the positive changes in lifestyle seen in this study. The positive changes in lifestyles noted were similar in the pre-diabetes and normoglycaemia group, and not different in the intervention areas (Huangdao and Jiaonan) from that in the non-intervention area (Jimo).

The small but significant increase in the ZSDS score in the women of both normoglycaemia and pre-diabetes groups may be a consequence of ageing and climacteric changes as the mean age of women was in their early fifties. Many diseases occur or further develop in middle-aged and elderly women related to the menopausal transition and ageing [27]. Assessing impact of screening or diagnosis on physical, mental and emotional well-being is complex because of the presence of many other influencing factors which cannot be isolated or controlled for. We observed the similar results that the 15D depression was slightly impaired in women, but not in men. In women with normoglycaemia the mean (SD) 15D depression scores were 0.986 (0.06) at baseline and 0.976 (0.08) at follow-up (p = 0.013); and 0.986 (0.06) and 0.972 (0.09) for women with pre-diabetes (p = 0.020). In men the scores were 0.993 (0.04) and 0.989 (0.05) for normoglycaemia (p > 0.05) and 0.991 (0.06) and 0.988 (0.06) for pre-diabetes (p > 0.05), respectively.

Our study has some limitations. We did not assess the HRQoL in individuals labelled as pre-diabetes within 1 or 2 weeks after the screening test results were disclosed, thus, the short-term psychological effects of screening cannot be determined. Based on the evidence from other published studies, the screening for type 2 diabetes had a minimal short-term effect on patients’ health status [20,22]. The follow-up rate was 57.3% and a selection bias can be argued. We compared the baseline characteristics of the participants with non-participants in the re-examination, and found that the non-responders (n = 1326) were slightly younger (50 vs. 52 years), more men (45.6% vs 39.1%), had lower BMI (24.4 vs. 24.9 kg/m^2^) and better HRQoL at baseline (mean 0.974 vs. 0.970) than the participants, but there was no differences in the ZSDS score, frequency in physical activity and fresh vegetable intake. The reasons for those individuals who did not participate in the second examination were: lost contact due to a city plan (7%), ill or deceased (3%), not arriving at the survey site because of various reasons such as busy, missed the date, not fasting or not being informed by the local community organisers (90%).

## Conclusions

This population-based diabetes screening program generated long-term positive changes toward a healthy lifestyle as measured by physical activity and vegetable intake for all the participants in spite of the labelling without adversely effect on the overall HRQoL and depression.
